# Nonlinear relationship between platelet count and 30-day in-hospital mortality in ICU acute respiratory failure patients: a multicenter retrospective cohort study

**DOI:** 10.1186/s40001-024-01909-1

**Published:** 2024-06-08

**Authors:** Pan Zhou, Qin-qin Guo, Fang-xi Wang, Li Zhou, Hao-fei Hu, Zhe Deng

**Affiliations:** 1 Department of Emergency Medicine, Shenzhen Second People’s Hospital/The First Affiliated Hospital of Shenzhen University Health Science Center, Shenzhen, 518035 China; 2 Department of Nephrology, Shenzhen Second People’s Hospital/The First Affiliated Hospital of Shenzhen University Health Science Center, Shenzhen, 518035 China

**Keywords:** Platelet count, Acute respiratory failure, 30-day in-hospital mortality, Nonlinear relationship, Multicenter study

## Abstract

**Background:**

Limited evidence exists regarding the link between platelet count and 30-day in-hospital mortality in acute respiratory failure (ARF) patients. Thus, this study aims to investigate this association among ICU patients experiencing acute respiratory failure.

**Methods:**

We conducted a retrospective cohort study across multiple centers, utilizing data from the US eICU-CRD v2.0 database covering 22,262 patients with ARF in the ICU from 2014 to 2015. Our aim was to investigate the correlation between platelet count and 30-day in-hospital mortality using binary logistic regression, subgroup analyses, and smooth curve fitting.

**Results:**

The 30-day in-hospital mortality rate was 19.73% (4393 out of 22,262), with a median platelet count of 213 × 10^9^/L. After adjusting for covariates, our analysis revealed an inverse association between platelet count and 30-day in-hospital mortality (OR = 0.99, 95% CI 0.99, 0.99). Subgroup analyses supported the robustness of these findings. Furthermore, a nonlinear relationship was identified between platelet count and 30-day in-hospital mortality, with the inflection point at 120 × 10^9^/L. Below the inflection point, the effect size (OR) was 0.89 (0.87, 0.91), indicating a significant association. However, beyond this point, the relationship was not statistically significant.

**Conclusion:**

This study establishes a clear negative association between platelet count and 30-day in-hospital mortality among ICU patients with ARF. Furthermore, we have identified a nonlinear relationship with saturation effects, indicating that among ICU patients with acute respiratory failure, the lowest 30-day in-hospital mortality rate occurs when the baseline platelet count is approximately 120 × 10^9^/L.

**Supplementary Information:**

The online version contains supplementary material available at 10.1186/s40001-024-01909-1.

## Introduction

Acute respiratory failure (ARF) is typically defined as the simultaneous occurrence of clinically significant hypoxia, hypercapnia, or both [1]. Approximately 784 cases per 100,000 hospitalized patients experience ARF [2]. One of the primary reasons adults admitted to the ICU is the need for tracheal intubation and mechanical ventilation support [3, 4]. Globally, around 20 million individuals require mechanical ventilation support annually [5]. While advancements like veno-venous extracorporeal membrane oxygenation (VV-ECMO) have enhanced survival rates [5–7], the mortality rate still ranges from 27 to 35% [8, 9]. Therefore, the identification of prognostic risk factors for ARF patients is crucial.

Platelets, produced by small megakaryocytes measuring 2–4 μm, circulate for approximately 7–10 days and play a primary role in hemostasis and coagulation [10, 11]. Beyond their established function in thrombosis, recent research has linked platelets to various physiological and pathological processes. Specifically, platelets may contribute to chronic obstructive pulmonary disease (COPD) development through activation, aggregation, and regulation of hypoxic signaling pathways [12]. Notably, platelets play a crucial role in both the progression and resolution of acute respiratory distress syndrome [13]. Moreover, in patients infected with COVID-19, microthrombosis mediated by platelet activation in alveolar capillaries is a significant mechanism leading to ARF [14–17]. Rohling et al. reported a slight increase in platelet count among patients with mild COVID-19 symptoms, while patients with severe COVID-19 exhibited a decrease in platelet count [18]. These findings underscore the multifaceted role of platelets in various physiological and pathological contexts, particularly in relation to respiratory diseases.

Currently, there is a lack of epidemiological evidence linking platelet count to the prognosis of patients with ARF. Specifically, within the population of ICU inpatients with unique physiological characteristics, no reports exist on this relationship. However, given that previous studies have indicated platelets as not only crucial hemostatic and coagulation indicators but also linked to various diseases, we hypothesize that the baseline platelet count before treatment may correlate with a poor prognosis in ICU-hospitalized ARF patients. Establishing this correlation could offer a fresh perspective for future research in this domain. To address this gap, utilizing high-quality data from multiple centers and large samples can provide reliable evidence for subsequent studies exploring similar themes.

Therefore, we analyzed the eICU-CRD v2.0 to investigate the relationship between baseline platelet count and 30-day in-hospital mortality in ICU patients with ARF.

## Methods

### Study design

This multicenter retrospective cohort study centers on platelet count as the independent variable and 30-day in-hospital mortality in patients with ARF as the dependent variable, categorized into death or survival.

### Data source

The eICU-CRD v2.0 is a substantial public database collaboratively established by Philips Healthcare and the Laboratory of Computational Physiology (LCP) at MIT. This database encompasses high-quality data from 200,859 patients admitted to intensive care units in 208 U.S. hospitals during 2014 and 2015. It includes diverse clinical information such as vital signs, nursing plans, disease severity, diagnosis, and treatment details [19]. Access to the data is available to the public upon registration, and the collection process adheres to the standards of the MIT Ethics Committee (No: 0403000206) and aligns with the 1964 Declaration of Helsinki.

### Study population

**Fig. 1:**
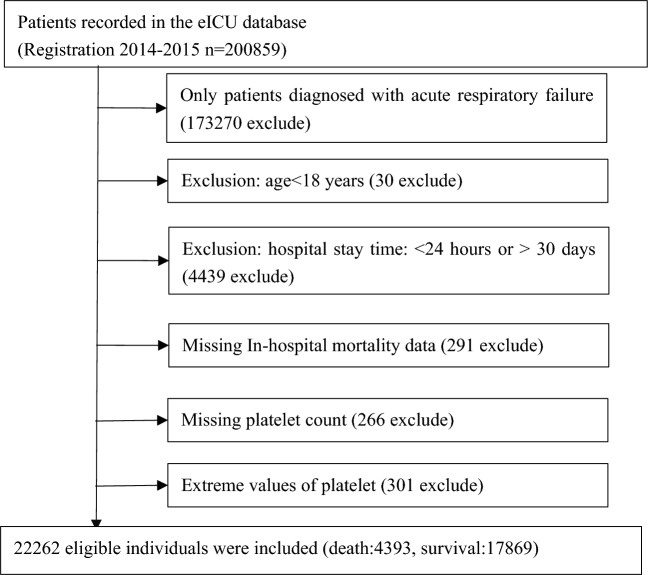
Flowchart of study participants

This study involved 22,262 eligible individuals, identified through specific exclusion criteria: (1) non-ARF patients (*n* = 173,270 excluded); (2) age < 18 years (*n* = 30 excluded); (3) ICU stay time < 24 h or > 30 days (*n* = 4439 excluded); (4) missing in-hospital mortality data (*n* = 291 excluded); (5) missing platelet count (*n* = 266 excluded); and (6) extreme platelet count values (three standard deviations above or below the mean) (*n* = 301 excluded). Following the application of these criteria, 22,262 participants remained for analysis. Figure 1 illustrates the participant selection process.

### Variables

The platelet count was first recorded as a continuous variable and later categorized into quartiles: Q1 (2.0–156.0 × 10^9^/L), Q2 (157.0–212.0 × 10^9^/L), Q3 (213.0–278.0 × 10^9^/L), and Q4 (279.0–569.0 × 10^9^/L).

### Covariates

Covariates were selected based on clinical experience and prior studies [20–24]. The included variables comprised as follows: (1) categorical variables such as sex, ethnicity, atrial fibrillation (AF), acute coronary syndrome (ACS), congestive heart failure (CHF), chronic kidney disease (CKD), chronic obstructive pulmonary disease (COPD), diabetes mellitus, gastrointestinal bleeding (GB), hypertension, sepsis, and the use of anti-platelet medications, anticoagulants, glucocorticoids, carbapenems, cephalosporins, vancomycin, and mechanical ventilation; (2) continuous variables including age, body mass index (BMI), hemoglobin concentration (Hb), serum creatinine (Scr), and Acute Physiology and Chronic Health Evaluation-IV score (APACHE-IV score). BMI was calculated as weight (kg) divided by height (m) squared (kg/m^2^). Baseline parameters were gathered within 24 h of ICU admission.

### Missing data processing

In this study, the percentages of participants with missing data for sex, BMI, Scr, and APACHE-IV score were 0.02%, 2.29%, 2.6%, and 13.39%, respectively. To address missing covariate data, multiple imputations were employed [25]. The imputation model included ethnicity, age, Hb, AF, ACS, CHF, CKD, COPD, diabetes mellitus, GB, hypertension, sepsis, use of anti-platelet medications, anticoagulants, glucocorticoids, carbapenems, cephalosporins, vancomycin, and mechanical ventilation. The missing data analysis assumption was based on the missing-at-random assumption (MAR) [26].

### Statistical analysis

Participants were divided into four groups according to their platelet count quartiles. Continuous variables are expressed as mean (standard deviation) for normally distributed data and median (range) for nonnormally distributed data. Categorical variables are presented as numbers and percentages. We utilized one-way analysis of variance for normally distributed data, the χ2 method for categorical variables, or the Kruskal–Wallis H test for skewed distribution to assess differences between platelet count groups.

#### To analyze the independent linear relationship of the platelet count and 30-day in-hospital mortality

After conducting collinearity screening (Table S1: no covariates were excluded), three models were developed using univariate and multivariate binary logistic regression, following the STROBE statement guidelines [27]. The objective was to explore the connection between platelet count and 30-day in-hospital mortality. The constructed models were as follows: (1) crude model (no covariate adjustments); (2) minimally adjusted model (model I: adjusted for sex, age, and ethnicity); and (3) fully adjusted model (model II: adjusted for sex, ethnicity, age, BMI, Hb, Scr, APACHE-IV score, AF, ACS, CHF, CKD, COPD, diabetes mellitus, GB, hypertension, sepsis, anti-platelet, anticoagulant, glucocorticoid, carbapenems, cephalosporins, vancomycin, and mechanical ventilation). Recorded effect sizes with 95% confidence intervals (95% CI) facilitated result interpretation. Covariate adjustments were applied if their inclusion led to a change in the odds ratio (OR) of 10% or more [27]. Subgroup analyses were performed to assess result robustness. Platelet count categorization into quartiles allowed the calculation of *P* for trend to test continuous variable outcomes and explore nonlinearity. Additionally, potential unmeasured confounding between platelet count and 30-day in-hospital mortality was examined using E-values.

#### Subgroup analysis

Subgroup analyses employed a stratified binary logistic regression model, covering various subgroups such as sex, ethnicity, AF, ACS, CHF, CKD, COPD, diabetes mellitus, GB, hypertension, sepsis, and mechanical ventilation. Each stratum, determined by the stratification factor, was adjusted for all relevant factors, including sex, ethnicity, age, BMI, Hb, Scr, APACHE-IV score, AF, ACS, CHF, CKD, COPD, diabetes mellitus, GB, hypertension, sepsis, anti-platelet, anticoagulant, glucocorticoid, carbapenems, cephalosporins, vancomycin, and mechanical ventilation. Additionally, interaction tests were conducted using the likelihood ratio test, comparing models with and without interaction terms [28, 29].

#### To analyze the nonlinear relationship of the platelet and 30-day in-hospital mortality

Utilizing binary logistic regression models can sometimes be limited in addressing nonlinearity. To address this concern, we applied generalized additive models (GAM) and employed smooth curve fitting (penalized spline method) to further investigate the nonlinear relationship between the platelet count and 30-day in-hospital mortality. In the presence of nonlinearity, our approach involved initially determining the inflection point through a recursive algorithm. Subsequently, we established a two-piece binary logistic regression model on either side of the inflection point [30]. The log-likelihood ratio test was then employed to identify the most appropriate model that describes the association between the platelet count and 30-day in-hospital mortality.

All results adhere to the STROBE statement [27]. Analyses were conducted using statistical software packages R (R Foundation)2 and EmpowerStats3 (X&Y Solutions, Inc., Boston, MA). Statistically significant findings were determined at a threshold of *P* < 0.05 (two sided).

## Results

### Characteristics of individuals

Table 1 presents the baseline characteristics of 22,262 participants stratified by platelet count quartiles. The distribution of participants differed significantly by sex and ethnicity across quartiles (both *P* < 0.05). Notably, the proportion of males decreased from 60.4% in Q1 to 44.8% in Q4, while the proportion of females increased from 39.6% in Q1 to 55.2% in Q4. Similarly, the distribution of ethnicity varied across quartiles (*P* = 0.012). Age, body mass index (BMI), hemoglobin (Hb), serum creatinine (Scr), and Acute Physiology and Chronic Health Evaluation-IV (APACHE-IV) score differed significantly across quartiles (all *P* < 0.001), with a trend of decreasing values from Q1 to Q4. The prevalence of comorbid conditions including atrial fibrillation (AF), acute coronary syndrome (ACS), congestive heart failure (CHF), chronic kidney disease (CKD), chronic obstructive pulmonary disease (COPD), diabetes mellitus, gastrointestinal bleeding (GB), hypertension, and sepsis also showed significant differences across quartiles (all *P* < 0.01), with a trend of decreasing prevalence from Q1 to Q4 for most conditions. Notably, the use of anti-platelets, carbapenems, and vancomycin, as well as the requirement for mechanical ventilation, demonstrated significant differences across quartiles (all *P* < 0.05). Mortality also varied significantly across quartiles, with the highest mortality in Q1 (25.27%) and lowest in Q3 (17.30%, *P* < 0.001). Additionally, platelet count is classified into four categories based on the degree of platelet reduction (Table S2). This study included a total of 22,262 participants, of whom 473 (2.13%) had very low platelet counts (< 50.0 × 10^9^/L), 1337 (6.01%) had intermediate-low platelet counts (50.0–100.0 × 10^9^/L), 3,134 (14.08%) had low platelet counts (100.0–150.0 × 10^9^/L), and 17,318 (77.78%) had normal platelet counts (≥ 150.0 × 10^9^/L). The 30-day in-hospital mortality rate decreased as platelet count increased, from 42.92% in the very low platelet count group to 17.92% in the normal platelet count group (*P* < 0.001).

**Table 1:** Baseline characteristics of participants (*N* = 22,262)

Platelet (× 10^9^/L)	Q1 (2.0–156.0)	Q2 (157.0–212.0)	Q3 (213.0–278.0)	Q4 (279.0–569.0)	*P*-value
Participants	5524	5578	5591	5569	
Sex					< 0.001
Male	3334 (60.4%)	3174 (56.9%)	2836 (50.7%)	2494 (44.8%)	
Female	2190 (39.6%)	2404 (43.1%)	2755 (49.3%)	3075 (55.2%)	
Ethnicity					0.012
Caucasian	4140 (74.95%)	4248 (76.16%)	4166 (74.51%)	4274 (76.75%)	
African American	606 (10.97%)	622 (11.15%)	693 (12.39%)	597 (10.72%)	
Hispanic	325 (5.88%)	302 (5.41%)	340 (6.08%)	309 (5.55%)	
Asian	78 (1.41%)	94 (1.69%)	77 (1.38%)	77 (1.38%)	
Other/unknown	375 (6.79%)	312 (5.59%)	315 (5.63%)	312 (5.60%)	
Age (years)	65.21 ± 15.07	65.25 ± 15.54	64.09 ± 16.05	63.03 ± 16.49	< 0.001
BMI (kg/m^2^)	28.14 ± 9.40	28.98 ± 9.70	28.90 ± 9.86	28.07 ± 9.53	< 0.001
Hb (g/dl)	11.17 ± 2.66	11.92 ± 2.51	11.99 ± 2.46	11.55 ± 2.51	< 0.001
Scr (mg/dl)	1.22 (0.85–2.07)	1.11 (0.80–1.77)	1.08 (0.80–1.65)	1.05 (0.75–1.65)	< 0.001
APACHE-IV score	77.83 ± 30.51	70.82 ± 28.71	70.55 ± 28.14	70.89 ± 27.70	< 0.001
Comorbid conditions					
AF	863 (15.62%)	831 (14.90%)	739 (13.22%)	656 (11.78%)	< 0.001
ACS	399 (7.22%)	503 (9.02%)	502 (8.98%)	466 (8.37%)	0.002
CHF	878 (15.89%)	992 (17.78%)	968 (17.31%)	875 (15.71%)	0.005
CKD	121 (2.19%)	138 (2.47%)	118 (2.11%)	87 (1.56%)	0.008
COPD	793 (14.36%)	1060 (19.00%)	1032 (18.46%)	927 (16.65%)	< 0.001
Diabetes mellitus	820 (14.84%)	929 (16.65%)	970 (17.35%)	883 (15.86%)	0.003
GB	447 (8.09%)	259 (4.64%)	243 (4.35%)	246 (4.42%)	< 0.001
Hypertension	868 (15.71%)	1081 (19.38%)	1094 (19.57%)	940 (16.88%)	< 0.001
Sepsis	1641 (29.71%)	1314 (23.56%)	1291 (23.09%)	1651 (29.65%)	< 0.001
Treatment					
Anti-platelet	335 (6.06%)	403 (7.22%)	420 (7.51%)	371 (6.66%)	0.014
Anticoagulant	213 (3.86%)	208 (3.73%)	217 (3.88%)	196 (3.52%)	0.737
Glucocorticoid	687 (12.44%)	749 (13.43%)	773 (13.83%)	760 (13.65%)	0.137
Carbapenems	149 (2.70%)	108 (1.94%)	127 (2.27%)	154 (2.77%)	0.014
Cephalosporins	515 (9.32%)	503 (9.02%)	497 (8.89%)	477 (8.57%)	0.570
Vancomycin	654 (11.84%)	626 (11.22%)	623 (11.14%)	730 (13.11%)	0.004
Mechanical ventilation	3709 (67.14%)	3603 (64.59%)	3599 (64.37%)	3620 (65.00%)	0.008
Mortality	1396 (25.27%)	1015 (18.20%)	967 (17.30%)	1015 (18.23%)	< 0.001

Categorical data are expressed as *n* (%)

Continuous data are expressed as mean ± SD or median (Q1–Q3)

BMI: body mass index; Hb: hemoglobin; Scr: creatinine; APACHE-IV score: Acute Physiology and Chronic Health Evaluation-IV score; AF: atrial fibrillation; ACS: acute coronary syndrome; CHF: congestive heart failure; CKD: chronic kidney disease; COPD: chronic obstructive pulmonary disease; GB: gastrointestinal bleeding; Mortality: 30-day in-hospital mortality

**Fig. 2:**
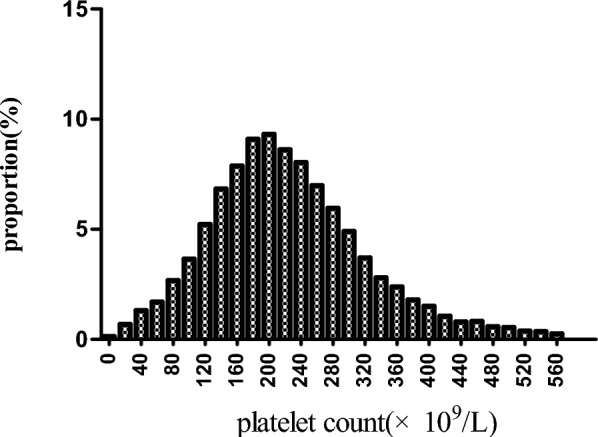
Distribution of platelet count. It displayed a nearly normal distribution ranging from 2.0 × 10^9^/L to 569.0 × 10^9^/L, with a median of 213.0 × 10^9^/L

**Fig. 3:**
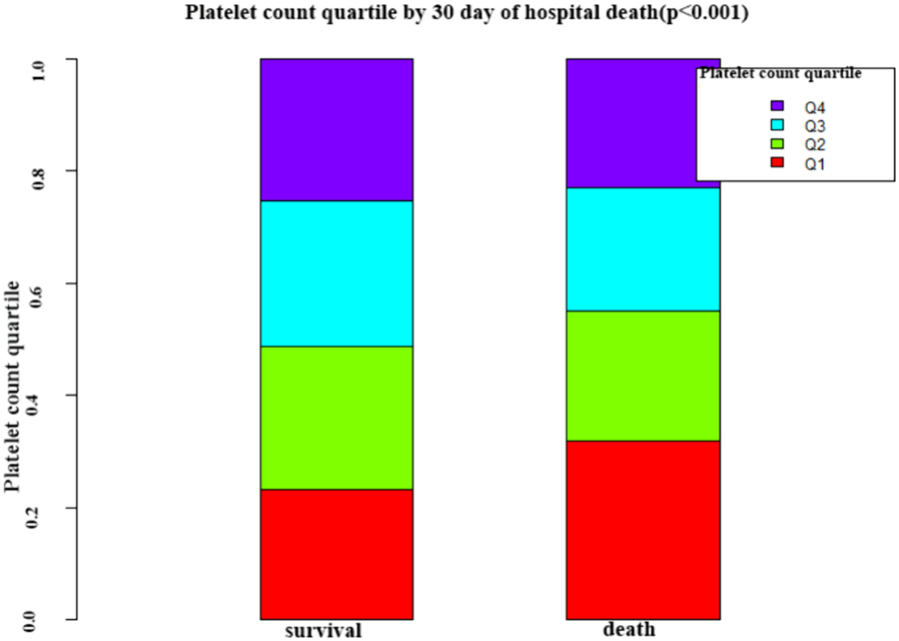
Platelet count levels in the survival and death groups. The figure illustrates lower platelet counts in the death group compared to the relatively higher platelet distribution in the survival group

Figure 2 depicts the distribution of platelet count, presenting an approximately normal distribution within the range of 2.0 × 10^9^/L to 569.0 × 10^9^/L, with a median of 213.0 × 10^9^/L. In Fig. 3, participants were categorized into two groups based on survival status. The platelet count distribution in the death group was notably lower, while the survival group exhibited a relatively higher platelet count.

### 30-Day in-hospital mortality results

**Fig. 4:**
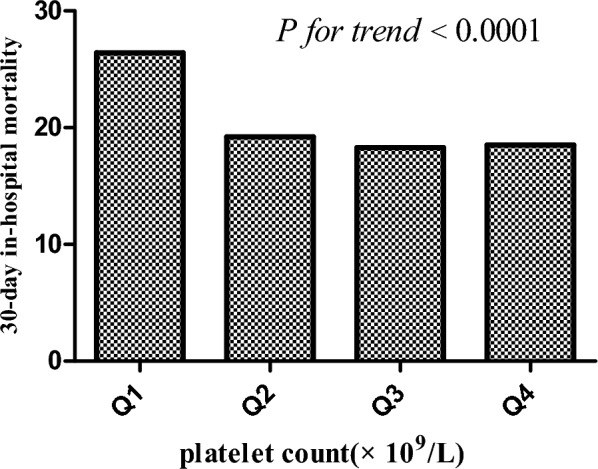
30-Day in-hospital mortality across platelet count quartiles

Table 2 presents the 30-day in-hospital mortality rates stratified by platelet count quartiles in a cohort of 22,262 critically ill patients. The overall incidence of mortality was 19.73% (4393/22262). A clear inverse association between platelet count quartiles and 30-day in-hospital mortality is evident. The mortality rates decreased with increasing platelet count quartiles, with a significant trend observed (*P* for trend < 0.0001). Specifically, the mortality rates for quartiles Q1, Q2, Q3, and Q4 were 25.27% (95% CI 24.13–26.42), 18.20% (95% CI 17.18–19.21), 17.30% (95% CI 16.30–18.29), and 17.51% (95% CI 16.51–18.52), respectively. A discernible downward trend in mortality rates is observed from lower to higher platelet count quartiles, as supported by the significant *P* for trend < 0.0001 (see Fig. 4). The findings of this study highlight the potential utility of platelet count as a prognostic marker for 30-day in-hospital mortality in ARF patients. The inverse relationship between platelet count and mortality rates suggests that platelet levels may serve as an important indicator of clinical outcomes in this patient population.

**Table 2:** Association between platelet count and 30-day in-hospital mortality in acute respiratory failure patients

Platelet count(× 10^9^/L)	Participants (*n*)	Mortality events	Incidence of mortality (95% CI) (%)
Total	22,262	4393	19.73 (19.21,20.26)
Q1	5524	1396	25.27 (24.13,26.42)
Q2	5578	1015	18.20 (17.18,19.21)
Q3	5591	967	17.30 (16.30,18.29)
Q4	5569	1015	17.51 (16.51,18.52)
*P* for trend			< 0.0001

### Univariate analyses results using a binary logistic regression model

Table S3 reports the results of univariate regression analysis of factors influencing 30-day in-hospital mortality. Older age was significantly associated with higher mortality (OR 1.02, 95% CI 1.02–1.02, *P* < 0.0001). Lower body mass index (OR 0.99, 95% CI 0.98–0.99, *P* < 0.001), hemoglobin (OR 0.93, 95% CI 0.92–0.95, *P* < 0.0001), platelet count (OR 1.00, 95% CI 1.00–1.00, *P* < 0.0001), and higher creatinine (OR 1.05, 95% CI 1.03–1.07, *P* < 0.0001) and APACHE-IV score (OR 1.02, 95% CI 1.02–1.03, *P* < 0.0001) also predicted higher mortality risk. AF (OR 1.73, 95% CI 1.59–1.89, *P* < 0.0001), ACS (OR 1.34, 95% CI 1.20–1.50, *P* < 0.0001), CKD (OR 1.38, 95% CI 1.12–1.71, *P* = 0.0028), GB (OR 1.65, 95% CI 1.45–1.89, *P* < 0.0001), and sepsis (OR 1.54, 95% CI 1.43–1.65, *P* < 0.0001) were associated with increased mortality. Chronic heart failure was not associated with mortality (*P* = 0.6945). The use of anticoagulants (OR 1.24, 95% CI 1.06–1.47, *P* = 0.0092), carbapenems (OR 1.83, 95% CI 1.52–2.21, *P* < 0.0001), vancomycin (OR 1.43, 95% CI 1.30–1.57, *P* < 0.0001), and mechanical ventilation (OR 1.77, 95% CI 1.64–1.90, *P* < 0.0001) were linked to higher mortality. Sex, ethnicity, hypertension, anti-platelets, glucocorticoids, and cephalosporins did not influence mortality (all *P* > 0.05).

### Multivariate analyses results using the binary logistic regression model

Table 3 delves into the association between platelet count and 30-day in-hospital mortality through crude, adjusted, and fully adjusted logistic regression models. In the crude model, a 10 × 10^9^/L increase in platelet count is linked to a 2% decrease in mortality rate (OR 0.98, 95% CI 0.98–0.99, *P* < 0.0001). In the minimally adjusted model (Model I), where adjustments were made solely for demographic variables (age, sex, and ethnicity), each 10 × 10^9^/L rise in platelet count corresponds to a 2% decrease in mortality rate (OR 0.98, 95% CI 0.98–0.99, *P* < 0.001). (OR 0.98, 95% CI 0.98–0.99, *P* < 0.0001). In the fully adjusted model (Model II), a 10 × 10^9^/L increase in platelet count is associated with a 1% decrease in mortality rate (OR = 0.99, 95% CI 0.99–0.99, *P* < 0.0001). Significantly, in all three models, the mortality risk of Q2, Q3, and Q4 was lower than that in Q1, and the dose–response relationship remained significant (all *P* for trend < 0.001). The study findings consistently demonstrate an independent and significant inverse association between platelet count and 30-day in-hospital mortality, with a graded protective effect observed across platelet count quartiles. Additionally, we computed an E-value to evaluate sensitivity to unmeasured confounding, which was determined to be 1.11. This value, exceeding the relative risk of unmeasured confounders and platelet count, indicates that unknown or unmeasured confounders had minimal impact on the relationship between platelet count and 30-day in-hospital mortality.

**Table 3:** Relationship between platelet and 30-day in-hospital mortality in different models

Exposure	Crude model (OR, 95%CI, *P*)	Model I (OR, 95%CI, *P*)	Model II (OR, 95%CI, *P*)
Platelet count	0.98 (0.98, 0.99) < 0.0001	0.98 (0.98, 0.99) < 0.0001	0.99 (0.99, 0.99) < 0.0001
Platelet count (quartile)			
Q1	Ref	Ref	Ref
Q2	0.66 (0.60, 0.72) < 0.0001	0.65 (0.60, 0.72) < 0.0001	0.77 (0.70, 0.85) < 0.0001
Q3	0.62 (0.56, 0.68) < 0.0001	0.63 (0.57, 0.69) < 0.0001	0.74 (0.67, 0.81) < 0.0001
Q4	0.66 (0.60, 0.72) < 0.0001	0.69 (0.62, 0.75) < 0.0001	0.77 (0.70, 0.85) < 0.0001
*P* for trend	< 0.0001	< 0.0001	< 0.0001

CI: confidence interval

Model I adjusted for age, sex, and ethnicity

Model II adjusted for adjusted age, sex, and ethnicity, BMI, Hb, Scr, APACHE-IV score, AF, ACS, CHF, CKD, COPD, Diabetes mellitus, GB, hypertension, sepsis, anti-platelet, anticoagulant, glucocorticoid, carbapenems, cephalosporins, vancomycin, and mechanical ventilation

### Subgroup analysis results

We designated sex, ethnicity, AF, ACS, CHF, CKD, COPD, diabetes mellitus, GB, hypertension, sepsis, and mechanical ventilation as predetermined effect modifiers. Our objective was to observe the stability of the association between platelet count and 30-day in-hospital mortality across various subgroups. Based on the results presented in Table 4, the interaction analysis and subgroup analysis revealed several important findings related to the association between various characteristics and 30-day in-hospital mortality. There were significant interactions between platelet count and sex (*P* for interaction = 0.0114), ethnicity (*P* for interaction = 0.0476), COPD (*P* for interaction = 0.0186), diabetes mellitus (*P* for interaction = 0.0198), and GB (*P* for interaction = 0.0010). Specifically, lower platelet count was significantly associated with higher mortality in female patients (OR 0.98, 95% CI 0.98–0.99, *P* < 0.0001) but not in male patients (OR 0.99, 95% CI 0.99–1.00, *P* = 0.0100). Caucasian patients had a significant association between lower platelet count and mortality (OR 0.99, 95% CI 0.99–0.99, *P* < 0.0001), while the association was not significant in African American and Asian patients. Among patients with COPD, lower platelet count was associated with increased mortality (OR 1.00, 95% CI 0.99–1.01, *P* = 0.9907) but not without COPD (OR 0.99, 95% CI 0.98–0.99, *P* < 0.0001). Similarly, the association was significant in patients without diabetes (OR 0.99, 95% CI 0.98–0.99, *P* < 0.0001) but not with diabetes (OR 1.00, 95% CI 0.99–1.01, *P* = 0.7982). Patients with GB had a stronger association between lower platelet count and mortality (OR 0.97, 95% CI 0.95–0.98, *P* < 0.0001) compared to those without GB. There were no significant interactions between platelet count and atrial fibrillation, acute coronary syndrome, congestive heart failure, chronic kidney disease, hypertension, sepsis, or mechanical ventilation (all *P* for interaction > 0.05). This nuanced understanding of the interplay between platelet count and mortality risk can ultimately contribute to improved patient outcomes and healthcare delivery.

**Table 4:** Results of interaction analysis and subgroup analysis

Characteristic	No of participants	OR 95% CI, *P*	*P* for interaction
Sex			0.0114
Male	11,839 (53.18%)	0.99 (0.99, 1.00) 0.0100	
Female	10,423 (46.82%)	0.98 (0.98, 0.99) < 0.0001	
Ethnicity			0.0476
Caucasian	16,828 (75.59%)	0.99 (0.99, 0.99) < 0.0001	
African American	2518 (11.31%)	0.99 (0.98, 1.00) 0.12451	
Hispanic	1276 (5.73%)	0.98 (0.97, 1.00) 0.0365	
Asian	326 (1.46%)	1.02 (0.99, 1.05) 0.2084	
Other/unknown	1314 (5.90%)	0.97 (0.96, 0.99) 0.0002	
AF			0.3525
No	19,173 (86.12%)	0.99 (0.98, 0.99) < 0.0001	
Yes	3089 (13.88%)	0.99 (0.98, 1.00) 0.1044	
ACS			0.5277
No	20,392 (91.60%)	0.99 (0.98, 0.99) < 0.0001	
Yes	1870 (8.40%)	0.99 (0.98, 1.00) 0.2099	
CHF			0.9799
No	18,549 (83.32%)	0.99 (0.99, 0.99) < 0.0001	
Yes	3713 (16.68%)	0.99 (0.98, 1.00) 0.0195	
CKD			0.4566
No	21,798 (97.92%)	0.99 (0.99, 0.99) < 0.0001	
Yes	464 (2.08%)	1.00 (0.97, 1.02) 0.9052	
COPD			0.0186
No	18,450 (82.88%)	0.99 (0.98, 0.99) < 0.0001	
Yes	3812 (17.12%)	1.00 (0.99, 1.01) 0.9907	
Diabetes mellitus			0.0198
No	18,660 (83.82%)	0.99 (0.98, 0.99) < 0.0001	
Yes	3602 (16.18%)	1.00 (0.99, 1.01) 0.7982	
GB			0.0010
No	21,067 (94.63%)	0.99 (0.99, 0.99) < 0.0001	
Yes	1195 (5.37%)	0.97 (0.95, 0.98) < 0.0001	
Hypertension			0.7408
No	18,279 (82.11%)	0.99 (0.98, 0.99) < 0.0001	
Yes	3983 (17.89%)	0.99 (0.98, 1.00) 0.0503	
Sepsis			0.6209
No	16,365 (73.51%)	0.99 (0.99, 0.99) < 0.0001	
Yes	5897 (26.49%)	0.99 (0.98, 0.99) < 0.0001	
Mechanical ventilation			0.9032
No	7731 (34.73%)	0.99 (0.98, 1.00) 0.0011	
Yes	14,531 (65.27%)	0.99 (0.98, 0.99) < 0.0001	

AF: atrial fibrillation; ACS: acute coronary syndrome; CHF: congestive heart failure; CKD: chronic kidney disease; COPD: chronic obstructive pulmonary disease; GB: gastrointestinal bleeding; Mortality: 30-day in-hospital mortality

### Addressing nonlinearity with generalized additive models (GAM)

**Fig. 5:**
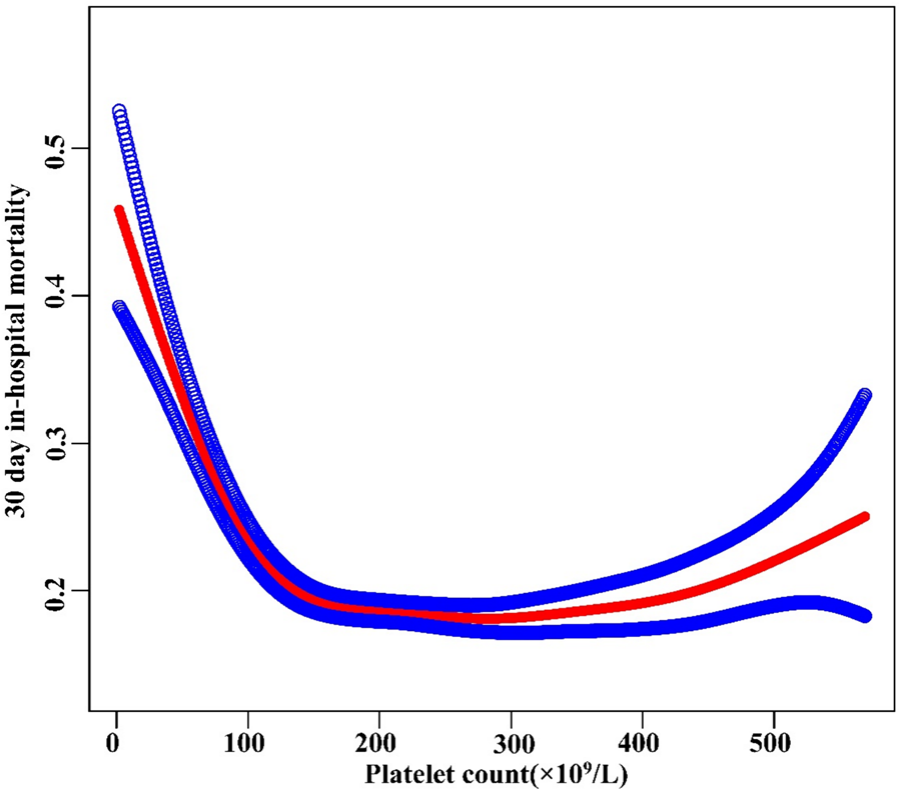
The nonlinear relationship between platelet count and 30-day in-hospital mortality. A nonlinear relationship was detected after adjusting for sex, ethnicity, age, BMI, Hb, Scr, APACHE-IV score, AF, ACS, CHF, CKD, COPD, Diabetes mellitus, GB, hypertension, sepsis, anti-platelet, anticoagulant, glucocorticoid, carbapenems, cephalosporins, vancomycin, and mechanical ventilation (Log-likelihood ratio test *P* < 0.001)

In this study, we employed GAM to explore potential nonlinearities in the relationship between platelet count and 30-day in-hospital mortality (refer to Fig. 5). Following adjustments for various factors such as sex, ethnicity, age, BMI, Hb, Scr, APACHE-IV score, AF, ACS, CHF, CKD, COPD, diabetes mellitus, GB, hypertension, sepsis, anti-platelet, anticoagulant, glucocorticoid, carbapenems, cephalosporins, vancomycin, and mechanical ventilation, a significant nonlinear association emerged (Log-likelihood ratio test *P* < 0.0001). Table 5 presents the results of a two-piecewise linear regression model examining the relationship between platelet count and 30-day in-hospital mortality. The inflection point of the platelet count was determined to be 120 × 10^9^/L. For patients with platelet counts less than or equal to 120 × 10^9^/L, each 10 × 10^9^/L increase in platelet count is significantly associated with a decreased mortality rate, with a ratio of 0.89 (95% CI 0.87–0.91, *P* < 0.0001). This suggests that higher platelet levels in this range were protective against short-term mortality. Conversely, for patients with platelet counts greater than 120 × 10^9^/L, there was no significant relationship between platelet count and mortality (OR: 1.00, 95% CI 1.00–1.00, *P* = 0.7057). This indicates that further increases in platelet levels did not provide additional survival benefits in this patient group. The log-likelihood ratio test yielded a highly significant result (*P* < 0.001), indicating that the two-piecewise regression model provided a significantly better fit to the data than the standard linear regression model.

**Table 5:** The result of the two-piecewise linear regression model

30-day in-hospital mortality	OR, 95%CI, *P*
Fitting model by standard linear regression	0.99 (0.99, 0.99) < 0.0001
Fitting model by two-piecewise regression	
Inflection point of platelet count	120 × 10^9^/L
≤ 120 × 10^9^/L	0.89 (0.87, 0.91) < 0.0001
> 120 × 10^9^/L	1.00 (1.00, 1.00) 0.7057
*P* for log-likelihood ratio test	< 0.001

## Discussion

This study employed multicenter eICU-CRD v2.0 data to investigate the connection between platelet count and 30-day in-hospital mortality among ICU patients with ARF. The results indicate that a higher platelet count is significantly associated with a reduced risk of mortality. Notably, this correlation is more pronounced in women, Caucasian patients, and those with COPD, diabetes, or GB. A threshold effect curve highlights distinct associations between platelet count and 30-day in-hospital mortality on either side of the inflection point. Below 120 × 10^9^/L, each 10 × 10^9^/L increase in platelet count is linked to an 11% decrease in the 30-day hospitalization mortality rate. However, beyond 120 × 10^9^/L, the elevation in platelet levels no longer significantly impacts mortality.

The link between platelet count and adverse outcomes in critically ill patients is gaining attention. Thachil et al. highlighted low platelet count as a common hematological issue and a risk factor for poor prognosis in ICU-treated patients [31, 32]. In a cohort study involving 215,098 ICU patients, Jonsson et al. found a connection between thrombocytopenia and increased mortality [33]. Anthon et al. conducted a prospective cohort study across 52 ICUs with 1166 patients, revealing that 43% of critically ill patients developed thrombocytopenia, resulting in worse outcomes, including higher mortality [34]. These studies emphasize platelet count as a crucial prognostic indicator for critically ill ICU patients. Moreover, in a retrospective study with 167 COVID-19 ICU patients, Zhu et al. discovered that thrombocytopenia not only correlated with respiratory function decline but also increased mortality in severe COVID-19 cases [35]. These studies all prompt us to pay attention to the relationship between thrombocytopenia and the prognosis of ARF. Despite this information, there is a noticeable gap in literature regarding the relationship between baseline platelet count and the 30-day risk of in-hospital mortality in ICU ARF patients, especially the saturation effect relationship. Prompted by these observations, we aimed to explore the correlation between baseline platelet levels and the 30-day in-hospital mortality risk in ICU ARF patients.

This study not only established an association between low baseline platelet count and increased mortality in ICU ARF patients but also identified a saturation threshold effect. Initially, the authors examined the linear relationship between baseline thrombocytopenia and the 30-day in-hospital death risk in ICU ARF patients through univariate and multivariate logistic regression analysis. A higher platelet count demonstrated a significant and independent correlation with lower 30-day mortality in a dose-dependent manner (*P* for trend 0.0001). Additionally, this relationship is more obvious in women, Caucasian patients and patients with COPD, diabetes or GB through subgroup risk analysis (*P* for interaction < 0.05). Furthermore, employing GAM and smooth curve fitting methods, the authors delved into the nonlinear relationship between platelet counts and 30-day in-hospital death in ARF patients. Clinically, this implies that among patients with severe thrombocytopenia, even modest increases in platelet count confer meaningful survival benefits. However, once the count surpasses approximately 120 × 10^9^/L, additional increments cease to impact prognosis. This nonlinear relationship provides a reference for clinical risk assessment, which is of significant importance. In summary, the two-piecewise regression model revealed a clinically informative inflection point in the platelet–mortality association.

Building on prior research, we propose that thrombocytopenia and increased 30-day in-hospital mortality share common underlying mechanisms. Firstly, platelets play a crucial role in hemostasis and coagulation [11]. Thrombocytopenia raises the risk of primary or secondary hemorrhage in vital organs, reducing overall survival rates, a particular concern in critical ICU settings. Secondly, thrombocytopenia serves as an indirect marker of heightened illness severity [31, 36–39]. Patients with lower platelet counts often require more intensive medical interventions, such as increased use of vasoactive drugs, renal replacement therapy, and mechanical ventilation. As confirmed by Table 1 in this study, higher platelet counts were associated with a younger, healthier demographic with fewer comorbidities. In contrast, the Q1 group exhibited elevated creatinine levels, higher APACHE-IV scores, and a greater incidence of complications like gastrointestinal bleeding, sepsis, and CKD, all indicative of an increased risk of mortality (all *P* < 0.05). Notably, invasive mechanical ventilation is linked to serious adverse events, and avoiding unnecessary tracheal intubation remains a primary goal of treating patients with acute hypoxic respiratory failure [40]. Consequently, patients with low platelet counts are prone to prolonged ICU hospitalization and increased mortality. Thirdly, platelets are recognized as the first responders in innate immunity, engaging with pathogens, including bacteria and viruses, via various surface receptors [41–43]. Operating as immune cells, platelets also interact with other immune entities like neutrophils, monocytes, dendritic cells, and lymphocytes [44]. Individuals with thrombocytopenia lose their ability to deposit fibrinogen, guide neutrophil migration, and block damaged vascular systems after diapause [13]. These factors suggest that patients with lower baseline platelet counts may experience compromised immunity, elevating their risk of mortality. The mentioned reasons likely contribute to the observed negative correlation between platelet count and 30-day in-hospital mortality on the left side of this study's inflection point. Conversely, on the right side of the inflection point, this relationship loses statistical significance, possibly due to worsening patient conditions and increased influence of other confounding factors. Therefore, the authors assert that maintaining an appropriate platelet count is crucial.

Our study has notable strengths that enhance the depth and reliability of our findings. The multicenter design and relatively large sample size significantly contribute to the generalizability of our results. The uniqueness of our research lies in its focus on ICU ARF patients, a population not extensively studied in this context before. Additionally, our exploration of nonlinearity adds a nuanced layer to our understanding of the relationship between platelet count and 30-day in-hospital mortality, surpassing previous studies' scope. Using multiple imputations to handle missing data ensures the mitigation of potential bias, maximizing statistical power. We further strengthen our findings through interaction and subgroup analysis and calculating E-values. Beyond academic significance, the practical implications of our study are also evident in the economic and accessible nature of platelet count as a common laboratory measure.

This work, however, has certain limitations. Firstly, our study focuses only on Americans and requires additional validation across different geographical and racial groups, and the study population consists of patients admitted to the ICU a decade ago, which may not fully reflect current clinical realities, necessitating further research. Secondly, the observational nature of our study design prevents establishing definitive causality. Despite this, we meticulously adjusted for confounding factors, ensuring robustness through subgroup analysis. The calculated E-value also suggests that unmeasured confounders are unlikely to significantly impact the results. Thirdly, the absence of acute respiratory failure (ARF) classification data and the etiology of ARF in our database hampers a detailed exploration of the relationship between platelet count and specific ARF subtypes. It is essential to note that our study exclusively included ICU-hospitalized ARF patients, and verification is needed for those not admitted to the ICU. These considerations emphasize the importance of incorporating more detailed variables in the design of future studies.

In summary, this study uncovers a negative and nonlinear correlation between platelet count and 30-day in-hospital mortality in ICU ARF patients. Importantly, a threshold effect is noted, signifying a significant negative association when the platelet count is ≤ 120 × 10^9^/L. However, when the platelet count surpasses 120 × 10^9^/L, this relationship loses statistical significance. These insights provide clinicians with valuable references for risk assessment, which is of significant importance.

### Supplementary Information

Supplementary Material 1. Table S1. Collinearity diagnostics steps. Table S2. Baseline characteristics of participants (*N* = 22,262). Table S3 Influencing factors of 30-day in-hospital mortality using univariate regression analysis.

## Data Availability

Data sharing not applicable to this article as no datasets were generated or analyzed during the current study. All data are sourced from the eICU-CRD v2.0 database.
