# Supplementary material for: Nonlinear relationship between platelet count and 30-day in-hospital mortality in ICU acute respiratory failure patients: a multicenter retrospective cohort study
Source: Eur J Med Res. 2024 Jun 8;29:312. doi: 10.1186/s40001-024-01909-1 (PMC11161993; doi:10.1186/s40001-024-01909-1)
Supplement: Supplementary file 1 — Supplementary Material 1. Table S1. Collinearity diagnostics steps. Table S2. Baseline characteristics of participants (N = 22,262). Table S3 Influencing factors of 30-day in-hospital mortality using univariate regression analysis. [file 40001_2024_1909_MOESM1_ESM.docx]

**Table S1. Collinearity diagnostics steps**

| **Variable** | **VIF** |
| --- | --- |
|  | **Step 1** |
| Sex | 1 |
| Ethnicity | 1 |
| Age(years) | 1.2 |
| BMI(kg/m^2^) | 1 |
| Hb(g/dl) | 1.1 |
| Scr(mg/dl) | 1.1 |
| APACHE-IV score | 1.2 |
| AF | 1.1 |
| ACS | 1.1 |
| CHF | 1.1 |
| CKD | 1 |
| COPD | 1.2 |
| Diabetes mellitus | 1.1 |
| GB | 1 |
| Hypertension | 1.1 |
| Sepsis | 1.1 |
| Anti-platelet | 1.2 |
| Anticoagulant | 1.1 |
| Glucocorticoid | 1.2 |
| Carbapenems | 1.1 |
| Cephalosporins | 1.2 |
| Vancomycin | 1.3 |
| Mechanical ventilation | 1.2 |

Abbreviation: VIF: variance inflation factor; VIF = 1/ (1-R^2^).

Note: The variables with VIF>5 will be regarded as collinear variables and cannot be included in the multiple regression model

BMI, body mass index; Hb, hemoglobin; Scr, creatinine; APACHE-IV score, Acute Physiology and Chronic Health Evaluation–IV score; AF, atrial fibrillation; ACS, acute coronary syndrome; CHF, congestive heart failure; CKD, chronic kidney disease; COPD, chronic obstructive pulmonary disease; GB, gastrointestinal bleeding; Mortality, 30-day in-hospital mortality

**Table S2. Baseline Characteristics of participants (N =22262)**

| Platelet(×10^9^/L) | Very low (<50.0) | Intermediate-low (50.0-100.0) | low (100.0-150.0) | normal (≥150.0) | *P*-value |
| --- | --- | --- | --- | --- | --- |
| Participants | 473 | 1337 | 3134 | 17318 |  |
| Sex |  |  |  |  | <0.001 |
| male | 264 (55.81%) | 803 (60.06%) | 1919 (61.23%) | 8853 (51.12%) |  |
| Female | 209 (44.19%) | 534 (39.94%) | 1215 (38.77%) | 8465 (48.88%) |  |
| Ethnicity |  |  |  |  | <0.001 |
| Caucasian | 347 (73.36%) | 997 (74.57%) | 2346 (74.86%) | 13138 (75.86%) |  |
| African American | 44 (9.30%) | 153 (11.44%) | 357 (11.39%) | 1964 (11.34%) |  |
| Hispanic | 23 (4.86%) | 76 (5.68%) | 193 (6.16%) | 984 (5.68%) |  |
| Asian | 7 (1.48%) | 11 (0.82%) | 49 (1.56%) | 259 (1.50%) |  |
| Other/unknown | 52 (10.99%) | 100 (7.48%) | 189 (6.03%) | 973 (5.62%) |  |
| Age(years) | 60.37 ± 15.16 | 63.17 ± 14.82 | 66.60 ± 14.99 | 64.20 ± 16.01 | <0.001 |
| BMI (kg/m^2^) | 27.35 ± 8.93 | 27.53 ± 9.33 | 28.27 ± 9.52 | 28.68 ± 9.69 | <0.001 |
| Hb(g/dl) | 9.40 ± 2.39 | 10.65 ± 2.64 | 11.50 ± 2.56 | 11.83 ± 2.51 | <0.001 |
| Scr(mg/dl) | 1.21 (0.81-2.11) | 1.32 (0.85-2.30) | 1.21 (0.86-2.06) | 1.09 (0.79-1.70) | <0.001 |
| APACHE-Ⅳ score | 85.21 ± 31.13 | 83.23 ± 31.48 | 75.31 ± 29.79 | 70.83 ± 28.21 | <0.001 |
| **Comorbid conditions** |  |  |  |  |  |
| AF | 65 (13.74%) | 215 (16.08%) | 495 (15.79%) | 2314 (13.36%) | <0.001 |
| ACS | 34 (7.19%) | 92 (6.88%) | 234 (7.47%) | 1510 (8.72%) | 0.013 |
| CHF | 35 (7.40%) | 203 (15.18%) | 547 (17.45%) | 2928 (16.91%) | <0.001 |
| CKD | 8 (1.69%) | 21 (1.57%) | 80 (2.55%) | 355 (2.05%) | 0.135 |
| COPD | 40 (8.46%) | 143 (10.70%) | 500 (15.95%) | 3129 (18.07%) | <0.001 |
| Diabetes mellitus | 50 (10.57%) | 177 (13.24%) | 502 (16.02%) | 2873 (16.59%) | <0.001 |
| GB | 53 (11.21%) | 171 (12.79%) | 197 (6.29%) | 774 (4.47%) | <0.001 |
| Hypertension | 46 (9.73%) | 162 (12.12%) | 547 (17.45%) | 3228 (18.64%) | <0.001 |
| Sepsis | 218 (46.09%) | 444 (33.21%) | 829 (26.45%) | 4406 (25.44%) | <0.001 |
| **Treatment** |  |  |  |  |  |
| Antiplatelet | 12 (2.54%) | 56 (4.19%) | 228 (7.28%) | 1233 (7.12%) | <0.001 |
| Anticoagulant | 6 (1.27%) | 33 (2.47%) | 146 (4.66%) | 649 (3.75%) | <0.001 |
| Glucocorticoid | 80 (16.91%) | 149 (11.14%) | 393 (12.54%) | 2347 (13.55%) | 0.004 |
| Carbapenems | 26 (5.50%) | 40 (2.99%) | 72 (2.30%) | 400 (2.31%) | <0.001 |
| Cephalosporins | 43 (9.09%) | 111 (8.30%) | 303 (9.67%) | 1535 (8.86%) | 0.417 |
| Vancomycin | 82 (17.34%) | 164 (12.27%) | 357 (11.39%) | 2030 (11.72%) | 0.002 |
| Mechanical ventilation | 317 (67.02%) | 917 (68.59%) | 2082 (66.43%) | 11215 (64.76%) | 0.012 |
| **Mortality** | 203 (42.92%) | 415 (31.04%) | 672 (21.44%) | 3103 (17.92%) | <0.001 |

Categorical data are expressed as n (%).

Continuous data are expressed as mean ± SD or median (Q1-Q3).

BMI, body mass index; Hb, hemoglobin; Scr, creatinine; APACHE-IV score, Acute Physiology and Chronic Health Evaluation-IV score; AF, atrial fibrillation; ACS, acute coronary syndrome; CHF, congestive heart failure; CKD, chronic kidney disease; COPD, chronic obstructive pulmonary disease; GB, gastrointestinal bleeding; Mortality, 30 day in-hospital mortality.

**Table S3 Influencing factors of 30-day in-hospital mortality using univariate regression analysis**

| **Variable** | **Statistic** | **OR95%CI** | ***P*** |
| --- | --- | --- | --- |
| Sex |  |  |  |
| male | 11839 (53.18%) | 1.0 |  |
| Female | 10423 (46.82%) | 0.99 (0.93, 1.06) | 0.7411 |
| Ethnicity |  |  |  |
| Caucasian | 16828 (75.59%) | 1.0 |  |
| African American | 2518 (11.31%) | 0.95 (0.86, 1.06) | 0.3651 |
| Hispanic | 1276 (5.73%) | 1.14 (0.99, 1.31) | 0.0619 |
| Asian | 326 (1.46%) | 1.15 (0.88, 1.49) | 0.3157 |
| Other/unknown | 1314 (5.90%) | 1.12 (0.98, 1.29) | 0.0937 |
| Age(years) | 64.39 ± 15.82 | 1.02 (1.02, 1.02) | <0.0001 |
| BMI(kg/m^2^) | 28.52 ± 9.63 | 0.99 (0.98, 0.99) | <0.001 |
| Hb(g/dl) | 11.66 ± 2.56 | 0.93 (0.92, 0.95) | <0.0001 |
| Scr(mg/dl) | 1.68 ± 1.80 | 1.05 (1.03, 1.07) | <0.0001 |
| APACHE-Ⅳ score | 72.51 ± 28.94 | 1.02 (1.02, 1.03) | <0.0001 |
| Platelet count | 223.64 ± 97.97 | 1.00 (1.00, 1.00) | <0.0001 |
| **Comorbid conditions** |  |  |  |
| AF |  |  |  |
| No | 19173 (86.12%) | 1.0 |  |
| Yes | 3089 (13.88%) | 1.73 (1.59, 1.89) | <0.0001 |
| ACS |  |  |  |
| No | 20392 (91.60%) | 1.0 |  |
| Yes | 1870 (8.40%) | 1.34 (1.20, 1.50) | <0.0001 |
| CHF |  |  |  |
| No | 18549 (83.32%) | 1.0 |  |
| Yes | 3713 (16.68%) | 0.98 (0.90, 1.07) | 0.6945 |
| CKD |  |  |  |
| No | 21798 (97.92%) | 1.0 |  |
| Yes | 464 (2.08%) | 1.38 (1.12, 1.71) | 0.0028 |
| COPD |  |  |  |
| No | 18450 (82.88%) | 1.0 |  |
| Yes | 3812 (17.12%) | 0.73 (0.67, 0.80) | <0.0001 |
| Diabetes mellitus |  |  |  |
| No | 18660 (83.82%) | 1.0 |  |
| Yes | 3602 (16.18%) | 1.10 (1.01, 1.20) | 0.0346 |
| GB |  |  |  |
| No | 21067 (94.63%) | 1.0 |  |
| Yes | 1195 (5.37%) | 1.65 (1.45, 1.89) | <0.0001 |
| Hypertension |  |  |  |
| No | 18279 (82.11%) | 1.0 |  |
| Yes | 3983 (17.89%) | 0.87 (0.80, 0.95) | 0.0021 |
| Sepsis |  |  |  |
| No | 16365 (73.51%) | 1.0 |  |
| Yes | 5897 (26.49%) | 1.54 (1.43, 1.65) | <0.0001 |
| **Treatment** |  |  |  |
| Anti-platelet |  |  |  |
| No | 20733 (93.13%) | 1.0 |  |
| Yes | 1529 (6.87%) | 1.09 (0.96, 1.24) | 0.1993 |
| Anticoagulant |  |  |  |
| No | 21428 (96.25%) | 1.0 |  |
| Yes | 834 (3.75%) | 1.24 (1.06, 1.47) | 0.0092 |
| Glucocorticoid |  |  |  |
| No | 19293 (86.66%) | 1.0 |  |
| Yes | 2969 (13.34%) | 0.92 (0.84, 1.02) | 0.1144 |
| Carbapenems |  |  |  |
| No | 21724 (97.58%) | 1.0 |  |
| Yes | 538 (2.42%) | 1.83 (1.52, 2.21) | <0.0001 |
| Cephalosporins |  |  |  |
| No | 20270 (91.05%) | 1.0 |  |
| Yes | 1992 (8.95%) | 0.94 (0.84, 1.06) | 0.3427 |
| Vancomycin |  |  |  |
| No | 19629 (88.17%) | 1.0 |  |
| Yes | 2633 (11.83%) | 1.43 (1.30, 1.57) | <0.0001 |
| Mechanical ventilation |  |  |  |
| No | 7731 (34.73%) | 1.0 |  |
| Yes | 14531 (65.27%) | 1.77 (1.64, 1.90) | <0.0001 |

BMI, body mass index; Hb, hemoglobin; Scr, creatinine; APACHE-IV score, Acute Physiology and Chronic Health Evaluation-IV score; AF, atrial fibrillation; ACS, acute coronary syndrome; CHF, congestive heart failure; CKD, chronic kidney disease; COPD, chronic obstructive pulmonary disease; GB, gastrointestinal bleeding.
